# Innovative unified impact of magnetite iron nanoparticles and quercetin on broiler chickens: performance, antioxidant and immune defense and controlling of *Clostridium perfringens* infection

**DOI:** 10.3389/fvets.2024.1474942

**Published:** 2024-11-07

**Authors:** Afaf Al-Nasser, Azza S. El-Demerdash, Doaa Ibrahim, Marwa I. Abd El-Hamid, Hanan S. Al-Khalaifah, Ola M. El-borady, Eman Shukry, Mona M. El-Azzouny, Mona S. Ibrahim, Shereen Badr, Nahla S. Elshater, Tamer Ahmed Ismail, Shorouk El Sayed

**Affiliations:** ^1^Environment and Life Sciences Research Center, Kuwait Institute for Scientific Research, Safat, Kuwait; ^2^Laboratory of Biotechnology, Department of Microbiology, Agriculture Research Center (ARC), Animal Health Research Institute (AHRI), Zagazig, Egypt; ^3^Department of Nutrition and Clinical Nutrition, Faculty of Veterinary Medicine, Zagazig University, Zagazig, Egypt; ^4^Department of Microbiology, Faculty of Veterinary Medicine, Zagazig University, Zagazig, Egypt; ^5^Institute of Nanoscience and Nanotechnology, Kafr Elsheikh University, Kafr Elsheikh, Egypt; ^6^Mansoura Provincial Lab, Department of Food Hygiene, Agriculture Research Center (ARC), Animal Health Research Institute (AHRI), Mansoura, Egypt; ^7^Department of Bacteriology, Animal Health Research Institute (AHRI), Zagazig Branch, Agriculture Research Center (ARC), Zagazig, Egypt; ^8^Department of Poultry Diseases, Mansoura Provincial Lab (AHRI-Mansoura), Animal Health Research Institute (AHRI), Agricultural Research Center (ARC), Mansoura, Egypt; ^9^Department of Clinical Pathology, Mansoura Branch, Animal Health Research Institute, Agricultural Research Center (ARC), Mansoura, Egypt; ^10^Reference Laboratory for Veterinary Quality Control on Poultry Production, Agriculture Research Center, Animal Health Research Institute, Giza, Egypt; ^11^Department of Clinical Laboratory Sciences, Turabah University College, Taif University, Taif, Saudi Arabia

**Keywords:** quercetin, magnetite, necrotic enteritis, antimicrobial peptide, intestinal antioxidant defense, inflammation

## Abstract

Necrotic enteritis caused by *Clostridium perfringens* (*C. perfringens*) is characterized by poor performance and higher mortality rates in poultry farms. Novel dietary intervention involving bioactive molecules loaded into smart magnetized nano-system with a potent antioxidant function (quercetin-loaded Fe_3_O_4_-NPs), was evaluated for their impact on growth performance, intestinal immune and antioxidant defenses, and resistance against *Clostridium perfringens* in a necrotic enteritis challenge model. Four experimental groups comprising a total of 200 one-day-old Ross 308 broiler chickens were fed different diets: a control basal diet, a diet supplemented with quercetin (300 mg/kg), a diet with Fe_3_O_4_-NPs (60 mg/kg), and a diet with quercetin-loaded Fe_3_O_4_-NPs (300 mg/kg). These groups were then challenged with *C. perfringens* during the grower period. Dietary inclusion of quercetin-loaded Fe_3_O_4_-NPs prominently reduced *C. perfringens* colonization and its associated virulence genes expression, which subsequently restored the impaired growth performance and intestinal histopathological changes in challenged broilers. Quercetin-loaded Fe_3_O_4_-NPs supplemented group displayed higher *Lactobacillus* and *Bifidobacterium* counts, upregulation of intestinal host defense antimicrobial peptides related genes (avian *β*-defensin 6 and 12) and downregulation of intestinal inflammatory regulated genes (Interleukin-1 beta, C-X-C motif chemokine ligand 8, tumor necrosis factor-*α*, chemokine C–C motif ligand 20, inducible nitric oxide synthase and cycloox-ygenase-2). Intestinal redox balance was boosted via upregulation of catalase, superoxide dismutase, glutathione peroxidase and heme Oxygenase 1 genes along with simultaneous decrease in hydrogen peroxide_,_ reactive oxygen species and malondialdehyde contents in groups fed quercetin-loaded Fe_3_O_4_-NPs. Overall, new nutritional intervention with quercetin-loaded Fe_3_O_4_-NPs impacted better immune and antioxidant defenses, attenuated *C. perfringens* induced necrotic enteritis and contributed to better performance in the challenged birds.

## 1 Introduction

*Clostridium perfringens* (*C. perfringens*) is part of the normal gut flora in poultry; however, multiple factors including contaminated environment, food contents and overcrowding can favor its intestinal colonization and lead to the bacterial overgrowth (1). Therefore, they could become pathogenic causing NE ranging from subclinical to devastating outbreaks. Necrotic enteritis caused by *C. perfringens* significantly impacts profitability of poultry industry, even in its subclinical form. The subclinical form is characterized by damage to the intestinal mucosal barriers, leading to impaired feed efficiency and growth performance (2). In contrast, the clinical form results in alterations in tight junction proteins, severe gastroenteritis with microbiota dysbiosis, and diarrhea, often associated with high mortality rates in birds (3). *C. perfringens* enteric infection gained public health concern for humans, not only as a foodborne infection transmitted through chicken meat consumption (1), but also due to the increase in its incidence because of eliminating antibiotics in poultry feed, which is aimed at combating the emergence of antibiotic-resistant bacteria (4–6). The alternative natural products from herbal, essential oils, organic acids, prebiotics and probiotics, have garnered have garnered significant research interest in the poultry industry as growth promoters and substitutes for antibiotics (7–11). However, their exact roles in driving a protective host immunity and resistance against pathogenic burdens still need to be explicitly defined (7, 12–14). Quercetin (2-(3,4-dihydroxyphenyl)-3,5,7-trihydroxy-4H-chromen-4-one) is one of the flavonoid components that can be easily obtained from natural sources like apple, broccoli, onion, pea nut and red onion etc. Several studies have highlighted its antibacterial, anti-inflammatory and antioxidant activities (15). Similarly, dietary inclusion of quercetin improved growth performance and nutrient digestibility of broiler chickens (16). Additionally, study involving broiler chickens fed diets supplemented with quercetin at doses of 0.5 and 1 g/kg of feed revealed that, although there was no increase, in the feed intake, there was a cumulative increase in feed conversion ratio (FCR) in a dose dependent manner. The study also noted an increased heart weight-to-body weight ratio and a decrease in lipid oxidation rate. The study also demonstrated improved antioxidative stability of breast meat, leading to an increased shelf life during storage (17).

It has been demonstrated that quercetin ameliorates the intestinal inflammation induced by lipopolysaccharides (LPS) in laying hens. This mitigating effect was attributed to quercetin’s immunomodulatory properties, which restore the gut microbial community and improve intestinal barrier function. The data revealed that animals fed diets supplemented with quercetin showed upregulation in the relative mRNA expression levels of tight junction associated genes, such as claudin-1 and occludin in both jejunum and ileum. Although feeding quercetin did not significantly affect *TNF-α* expression, it resulted in upregulation of IL-4 gene expression and downregulation of proinflammatory-related genes in the quercetin-supplemented group. There was a difference in the relative mRNA expression of the inflammatory signature between ileum and jejunum. This variation may be attributed to differences in quercetin bioavailability and absorption in different parts of the intestine (18). The uncontrolled use of antibiotics as feed additives has been reported to not only cause the emergence of antibiotics resistance bacteria, but also the presence of drug residues in meat products, which can affect human health (19). Therefore, iron oxide nanoparticles have been investigated as a source of iron in poultry feed due to their high bioavailability, which helps to reduce the cost compared to conventional inorganic sources (20). In addition to their low toxicity, iron oxide nanoparticles, particularly those that are biologically synthesized, offer further advantages (21). The antimicrobial activity of iron oxide nanoparticles against *Staphylococcus aureus* and *Pseudomonas aeruginosa* isolated from poultry feed has been reported (21). The promising antimicrobial effects of iron oxide and its role as an iron source will reduce the cost of using commercial feed additives. However, the precise mechanism by which iron oxide enhances resistance to *C. perfringens* infection in broiler chickens has yet to be fully explored. Therefore, our study aimed to introduce new nanoliposomes formula combining iron and quercetin as feed additive in broilers’ diets with the goal of alleviating inflammatory necrotic enteritis (NE) caused by *C. perfringens*. The study showed whether this ameliorative effect could be associated with a promotion of poultry’s performance, immunity, barrier defense, antioxidant system or restore gut microbiota. Such effects could, in turn, prevent systemic inflammation and promote antioxidant stability in broiler meat.

## 2 Materials and methods

### 2.1 Preparation of quercetin-loaded magnetite (Fe_3_O_4_) nanoparticles

The co-precipitation method was used to prepare magnetite (Fe_3_O_4_) nanoparticles (22). Briefly, 500 g of iron salt solution containing FeCl_2_.4H_2_O (30 mmoL, Oxford, India), and FeCl_3_.6H_2_O (60 mmoL, Oxford, India) was prepared and mixed in an oxygen-free reactor and then, NaOH solution (0.8 M, Sigma-Aldrich, Germany) was added drop wise to the prepared iron salt solution. A brownish black precipitate was formed indicating Fe_3_O_4_ NPs formation. The powder was collected by centrifuging, washed with deionized water, and dried in oven at 80°C for 3 h.

**Figure 1:**
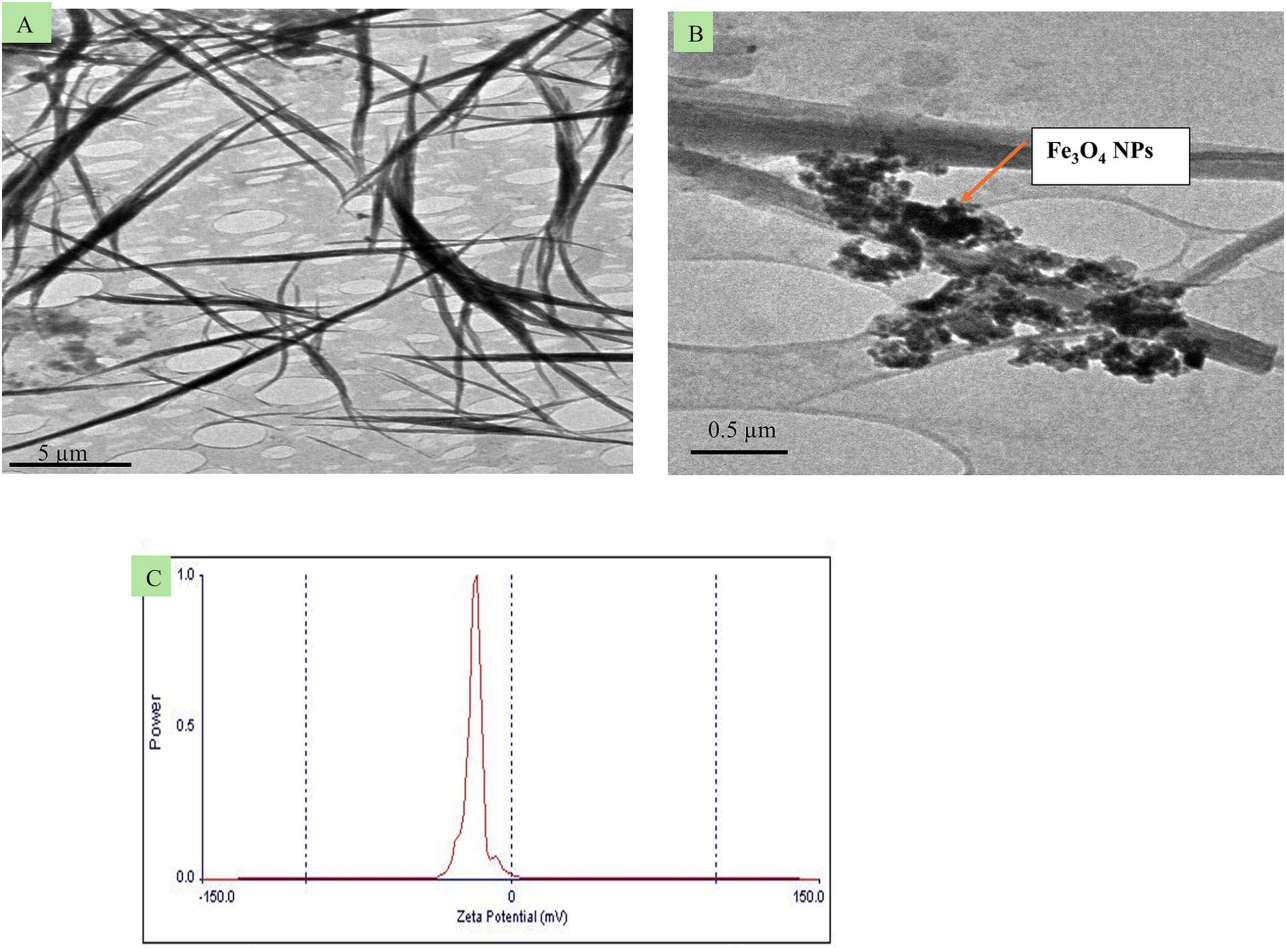
Transmission electron microscopy images **(A,B)** from different spots of the newly formulated quercetin-loaded magnetite (quercetin-loaded; Fe_3_O_4_-NPs), and **C**, its zeta potential.

To prepare quercetin-loaded Fe_3_O_4_ NPs emulsion, the emulsification-solvent technique (O/W) using a probe ultrasonicator was employed. First, 0.006 gm of the previously Fe_3_O_4_ NPs was dissolved in 20 mL of absolute ethanol (Merck, Germany NaOH, AppliChem, Panreac, ITW companies, Germany). The mixture was sonicated for 1 h in water path sonicator. Then, 0.02 gm of quercetin powder (5 mg/mL, Sigma-Aldrich, Missouri, United States), was added to 4 mL of the previously prepared Fe_3_O_4_ NPs in ethanol solution (oil phase). The resulting solution was added dropwise to 100 mL of deionized water in a clean conical flask drop by drop using a medical syringe (5 mL) over 5 min. The mixture was stirred at 1000 rpm to ensure proper mixing. The morphology of the newly formulated (quercetin-loaded Fe_3_O_4_-NPs) was carried out using transmission electron microscopy (JOEL JEM-2010) operated with a 200 kV coupled acceleration voltage to the Gatan Erlangshen ES500 digital camera model (Figures 1A,B). The surface charging behavior in contact with water-based electrolytes was assessed by measuring the zeta potential of the prepared sample using a Malvern Zetasizer (Nano ZS-90) set to 25°C (Figure 1C). High-resolution transmission electron microscopy (HR-TEM) analysis revealed that the quercetin-loaded Fe₃O₄ nanoparticles (NPs) have wire-like structures with diameters ranging from 50 to 90 nm and lengths extending into the micrometer range. Additionally, agglomeration of spherical-shaped nanoparticles with sizes less than 50 nm was observed due to the Fe_3_O_4_ NPs. The prepared nanoemulsion exhibited a negative zeta potential of −16 ± 11 mV (Figure 1C), which is sufficient to prevent nanoparticle aggregation.

### 2.2 Birds and experimental protocol

A total of 200 one-day-old broiler chickens (Ross 308) was purchased from the commercial poultry hatchery. Birds were weighed before beginning the experimental process and divided into 4 separate groups (*n* = 50/group). The four groups were assigned as following; G1: supplemented with conventional basal diet defined as control group, G2: supplemented with basal diet containing Fe_3_O_4_-NPs alone (60 mg/kg diet), G3: supplemented with basal diet containing quercetin alone (300 mg/ kg diet) and G4: supplemented with basal diet containing the formulated Fe_3_O_4_-NPs loaded quercetin (300 mg/kg diet), with the iron concentration being 60 mg in the prepared nano mixture. Antibiotic free diets for starter, grower and finisher periods were prepared as stated in Table 1. All birds continued to receive these formulated diets during the whole study period (38 days) and they were raised according to Ross Broiler Management Guide (23). They were housed in hygienic conditions and had unlimited access to water and food during the study period. According to the Association of Official Analytical Chemists (24), all formulated diets underwent chemical analysis for moisture, crude protein, ether extract and crude fiber. All groups were orally challenged with 1×10^8^ colony forming units (CFUs) of *C. perfringens* strain for three successive days starting at 21st day of the experimental period.

**Table 1:** The basal diets’ ingredients and nutrients composition.

Item	Starter (1–10 days)	Grower (11–20 days)	Finisher (21–38 days)
Ingredient (%)			
Soybean meal (47.9%)	34.5	30.61	25.9
Yellow corn	59.11	61.85	65.81
Soybean oil	1.90	3.12	3.87
Calcium carbonate	1.40	1.40	1.40
Dicalcium phosphate	1.50	1.50	1.50
Choline chloride	0.20	0.20	0.20
Common salt	0.40	0.40	0.40
Anti-mycotoxin	0.10	0.10	0.10
L-Lysine HCL (Lysin, 78%)	0.34	0.32	0.32
DL-Methionine (Methionine, 99%)	0.25	0.20	0.20
Premix*	0.30	0.30	0.30
Nutrient composition			
Lysine (%)	1.52	1.29	1.21
Methionine (%)	0.61	0.52	0.52
Available phosphorus (%)	0.49	0.51	0.52
Calcium (%)	1.20	1.18	1.15
Ether extract (%)	4.29	5.59	6.58
Crude fiber (%)	2.52	2.54	2.45
Crude protein (%)	23.06	21.48	19.53
Metabolizable energy (kcal/kg)	3,108	3,106	3,201

*Vitamin premix supplied per kilogram of diet: I (iodide), 1.5 mg; Se (selenate), 0.45 mg; Mn (sulfate and oxide), 100 mg; Cu (sulfate), 15 mg; Fe (sulfate), 60 mg; Zn (sulfate and oxide), 120 mg; thiamine, 6 mg; pantothenate, pyridoxine, 6 mg; 15 mg; biotin, 300 μg; niacin, 50 mg; tocopherol acetate, 75 mg; retinol, 12.000 IU; vitB12, 18 μg; riboflavin, 7 mg; folate, 4 mg; and cholecalciferol, 8,000 IU.

### 2.3 Bacterial strain preparation

A field-isolated *C. perfringens* strain from NE cases from commercial broiler chickens flock in Sharkia Governorate was used in the challenge model. This strain was phenotypically identified according to conventional microbiological techniques and confirmed to be type A using a PCR assay (19). The strain was preserved at −80°C until use. Prior to injection, it was cultured on tryptose sulfite cycloserine agar (Oxoid, Basingstoke, United Kingdom) plates at 37°C for 18 h. A bacterial suspension of 1 × 10^8^ CFU/mL was then prepared and used for oral gavage of each bird at the time of the challenge, following the described experimental protocol.

### 2.4 Growth performance evaluation

Body weight (BW) and feed intake (FI) were measured on the first day and at the end of the study period to evaluate the body weight gain (BWG), FCR [feed intake(g/bird)/weight gain (g/bird)] as previously mentioned (10) Moreover, clinical signs and mortality rates were recorded throughout the experimental period. Deceased birds were necropsied and scored for NE associated intestinal gross lesions in line with the criteria established earlier (19).

### 2.5 Quantitative estimation of microbiota and *Clostridium perfringens* using real-time PCR

Quantification of *Lactobacillus* and *Bifidobacterium* species and *Enterobacteriaceae* populations at the end of experimental period and that of *C. perfringens* at 7 and 14 days post challenge were performed using quantitative real-time PCR (RT-PCR) technique. Briefly, DNA was extracted from intestinal contents via commercial QIAamp DNA stool kit (Qiagen, Germany) consistent with the manufacturer’s endorsements and then DNA concentration was measured via NanoDropTM 2000 spectrophotometer (Thermo Fisher Scientific Inc., United States). After that, *C. perfringens* populations were enumerated, in triplicates, utilizing SYBR-Green based RT-PCR method carried out on Stratagene MX3005P RT-PCR instrument via SYBR Premix Ex Taq™ kit (TaKaRa, Japan). For generating standard curves used for RT-PCR analysis, 10-fold serial dilutions of DNA extracted from pure cultures of the investigated bacterial species were performed. The copies of examined bacterial species were estimated in terms of log_10_ CFU per gram of the analyzed sample. The primer sequences used for quantification of bacterial species are mentioned in Table 2.

**Table 2:** Primer sequences used for quantitative PCR assays.

Encoding gene	Primer sequence (5′-3′)	Accession No./Reference
Inflammatory and stress related genes		
*IL-1β*	F: GCTCTACATGTCGTGTGTGATGAG R: TGTCGATGTCCCGCATGA	NM_204,524
*TNF-α*	F- CCCCTACCCTGTCCCACAA R- ACTGCGGAGGGTTCATTCC	XM_046900549.1
*CCL20*	F: AGGCAGCGAAGGAGCAC R: GCAGAGAAGCCAAAATCAAAC	NM_204438
*CXCL8*	F: GCCCTCCTCCTGGTTTCAG R: TGGCACCGCAGCTCATT	AJ009800
*IL-10*	F: GCTGAGGGTGAAGTTTGAGG R: AGACTGGCAGCCAAAGGTC	XM_025143715.1
*CYP17A1*	F: GCTGAAGAAGGGGAAGGCT R: GAAGGAGAGGGGCAGTG	(61)
*COX-2*	F: TGTCCTTTCACTGCTTTCCAT R: TTCCATTGCTGTGTTTGAGGT	NM_0,011,67718.1
*iNOS*	F: AGGCCAAACATCCTGGAGGTC R: TCATAGAGACGCTGCTGCCAG	(62)
*Casapase-1*	F: ACATATACCAGCCACGGGAGA R: CATTGTAGCCCAGCCCTTCT	(63)
*Casapase-3*	F: TGGTGGAGGTGGAGGAGC R: GTTTCTCTGTATCTTGAAGCACCA	(63)
Antimicrobial peptides defense genes		
*AVBD6*	F: GCCCTACTTTTCCAGCCCTATT R: GGCCCAGGAATGCAGACA	NM 001001193.1
*AVBD12*	F: TGTAACCACGACAGGGGATTG R: GGGAGTTGGTGACAGAGGTTT	NM 001001607.2
Antioxidant genes		
*GSH-Px*	F: AACCAATTCGGGCACCAG R: CCGTTCACCTCGCACTTCTC	HM590226
*SOD*	F: GGCAATGTGACTGCAAAGGG R: CCCCTCTACCCAGGTCATCA	NM_205064.1
*Catalase*	F: GGGGAGCTGTTTACTGCAAG R: GGGGAGCTGTTTACTGCAAG	NM_001031215.2
*NQO1*	F: TCGCCGAGCAGAAGAAGATTGAAG R: CGGTGGTGAGTGACAGCATGG	NM_001277620.1
*HO-1*	F: AAGAGCCAGGAGAACGGTCA R: AAGAGCCAGGAGAACGGTCA	NM_205344
House keeping		
*GAPDH*	F: CAACCCCCAATGTCTCTGTT R: TCAGCAGCAGCCTTCACTAC	NM205518
16S rRNA of bacterial species		
***C. perfringens***	F: CGCATAACGTTGAAAGATGG R: CCTTGGTAGGCCGTTACCC	(64)
*Bifidobacterium* species	F: TCGCGTCYGGTGTGAAAG R: CCACATCCAGCRTCCAC	(65)
*Enterobacteriaceae*	F: CATTGACGTTACCCGCAGAAGAAGC R: CTCTACGAGACTCAAGCTTGC	(66)
*Lactobacillus* species	F: AGCAGTAGGGAATCTTCCA R: CACCGCTACACATGGAG	(67)
*C. perfringens* virulence genes		
*cnaA*	F: GGTGGATGGGCAACATTTAC R: CCTTGCTTGGATTCACCAGT	(68)
*cpe*	F: ATAGATAAAGGAGATGGTTGGA R: CCATATTCTACAGATGCTTGTA	(68)
*netB*	F: ACCGCTTCACATAAAGGTTGG R: TCAGGCCATTTCATTTTTCCGT	(68)
*nagH*	F: TCATGGAGAATATATTGGGGTTA TCCACTCAACACCATTCATAG	(68)
*nanI*	F: AAGGTAAACAATCTAGTGCTGT R: TCTATTATCATTTGGAGATTCTC	(68)
*nanJ*	F: TGTTTATAAAACACAACCAGTAG R: CATCTATAGAAGCTAAAACCGT	(68)
*16S rRNA*	F: GGGGGTTTCAACACCTCC R: GCAAGGGATATCAAGTGT	(69)

Interleukin (*IL*)-*1β*, *IL*-6 and *IL*-*10*, tumor necrosis factor-α (*TNF*-α), C-X-C motif chemokine ligand 8 (*CXCL8*), cytochrome P450, family 17, subfamily A, polypeptide 1 (CYP17A1), chemokine C–C motif ligand 20 or also known as macrophage inflammatory proteins −3 α (*CCL20*), Avian β-defensin 6 and 12 (*AvBD6* and *AvBD12*), Catalase (*CAT*), superoxide dismutase (*SOD*), glutathione peroxidase (*GSH*-*Px*), heme oxygenase-1 (*HO*-*1*), NAD(P)H dehydrogenase quinone 1 (*NQO1*), Inducible nitric oxide synthase (*iNOS*), cycloox-ygenase-2 (*COX-2*) glyceraldahyde-3-phosphate dehydrogenase (*GAPDH*), *C. perfringens* enterotoxin (*CPE*), collagen adhesin protein (*CNA*), necrotic enteritis B-like toxin (*NetB*), three different sialidases, named *NanH*, *NanI*, and *NanJ*, *nagH* (hyaluronidase or μ-toxin).

### 2.6 Quantitative reverse transcriptase polymerase chain reaction (qRT-PCR) for evaluation of intestinal immune response, barrier functions, antioxidant status and *Clostridium perfringens* virulence related genes expression

Intestinal and breast meat specimens were collected and frozen in liquid nitrogen for evaluation of antimicrobial peptide defense, inflammatory response, antioxidant status and *C. perfringens* virulence associated genes expression using qRT-PCR techniques. RNA isolation was performed along with the manufacturer’s directions using QIAamp RNeasy Mini kit (Qiagen GmbH, Hilden, Germany) and then RNA concentration and purification were assessed using Nanodrops 2000 spectrophotometer (Thermo Fisher Scientific Inc., Waltham, MA, United States) at optical densities of 260 and 280 nm. One-step qRT-PCR was performed as per the manufacture’s guidelines of Stratagene™ Mx3005P real-time quantitative polymerase chain reaction system (Thermo Fisher Scientific, Waltham, MA, United States) and SYBR Green based real-time PCR Kit (Qiagen, Hilden, Germany). The primer sequences used for all qRT-PCR assays are stated in Table 2.

### 2.7 Biochemical analysis of oxidative traits of breast meat

Meat samples from all groups were collected at the end of the experimental period (38 days) for evaluation of oxidative traits of breast meat including malondialdehyde (MDA), total antioxidant capacity (T-AOC), reactive oxygen species (ROS) and hydrogen peroxide (H_2_O_2_). The reduction of H_2_O_2_ concentration was performed at 25°C at 240 nm using Halo DB-2O double beam spectrophotometer (Dynamica, Australia). The MDA as a secondary lipid oxidation product formed by hydrolysis of lipid hydroperoxides given in nMoL/g of meat tissues was measured as previously mentioned (17). In addition, meat filtrates were used for measurement of T-AOC according to manufacturer’s instruction of commercial assay kits (Sigma-Aldrich, MAK187).

### 2.8 Measurement of hematological and immune-response related parameters

Blood samples from the wing vein were collected from each replicate in two sections. One on heparinized tube to prevent clotting for estimating red blood cells (RBCs) count, hemoglobin (Hb) concentration and isolation of peripheral blood mononuclear cells for evaluating phagocytosis and killing assay. The second section was collected in a clot activator vacutainer tube to allow clotting and centrifuged for 10 min at 3,000 rpm for separation of serum for evaluation of lysozyme activity and nitric oxide production.

The count of RBCs was assessed via a Neubauer hemocytometer (Sigma-Aldrich, Germany) and Hb concentrations were calculated using the cyanomethemoglobin colorimeteric method.

For evaluation of phagocytosis and candidacidal activity, the peripheral blood mononuclear cells were separated by a modified method Sun et al. (25). Briefly, 2 mL of heparinized blood was diluted 1:1 with 1X of phosphate-buffered saline (PBS), the diluted blood was layered on the surface of 2 mL of Histopaque 1,083 (Sigma-Aldrich) and then centrifuged at 500 XG for 20 min. The interface between the Histopaque and the first layer was further isolated. After washing with 1X of PBS 3 times, the cell pellets were re-suspended in complete RPMI 1640 medium (Invitrogen, Cat#11875119) containing 10% fetal bovine serum (HyClone, Logan, UT), 100 U/mL of penicillin and 100 μg/mL of streptomycin. The total number of the cells and their viability were determined using the hemocytometer and trypan blue exclusion method.

The phagocytosis assay was performed as previously mentioned with modifications (26). Briefly, 0.5 McFarland standard of yeast suspension containing 10^6^ cells /mL was prepared. Then, 0.5 mL of the yeast suspension was added to a tube containing 1 mL of MO/MQ cells suspension consisting of 0.5 mL of 1X PBS and 0.5 mL of RPMI 1640 media with 100 μL of activated fetal calf serum. After a 2 h incubation period, different slide smears from the cell suspensions containing yeast were prepared, stained with Giemsa stain and examined under microscope using oil emersion lens. Around 200 cells in different fields were scanned for evaluation of the percentage of phagocytic activity determined by counting the number of macrophages with ingested intracellular yeast cells, while the candidacidal activity was indicated by the number of digested or killed yeast “Ghost cells” present per 100 cells of phagocytosed candida.

Lysozyme activity in serum samples was evaluated according to the method mentioned formerly (27). Briefly, 1.9 mL of *Micrococcus Lysideikticus* (0.2 mg/mL) suspension in sodium phosphate buffer (pH = 6.2) was added to 100 μL of avian serum. Then, the reaction was incubated at 25° C for 0.5 and 4.5 min before measuring the absorbance on turbidimeter at 530 nm in both time intervals. The lysozyme activity was indicated by the amount of sample required to reduce the absorbance by 0.001 per minute indicating the increase in lysis of *Micrococcus lysideikticus*. Serum NO contents were evaluated as previously mentioned (19) using the commercial kits (Jiancheng Biotechnology Institute, Nanjing, China).

### 2.9 Histopathological examination

Samples were taken from intestinal tissues at the end of the experimental period and fixed in 10% neutral buffered formalin and the fixed tissue sections were then processed for routine histopathological procedures. The tissues were subsequently embedded in paraffin wax for sectioning, stained with hematoxicillin-eosin and examined under the light microscope. Died birds were necropsied and subjected to metric evaluation of histological alterations in ileal tissues consistent with the criteria established formerly (28), in which the impact factor (IF) ranged from 1 to 3 with 3 denoting sever lesions. Furthermore, the intensity of each lesion (S) was scored within the range of 0 to 3; no lesion, up to 25% alteration, 25 to 50% alteration and more than 50% alteration, respectively. Histopathological alterations were evaluated in relation to the following formula; *Σ*(IF*S) (28).

### 2.10 Statistical analysis

Statistical variance was analyzed via SPSS 27.0 software (IBM Corp., Chicago, United States) using one way analysis of variance (ANOVA). To determine statistical variations among the evaluated means, Tukey’s test was utilized, where *p* values lower than 0.05 implied that the experimental model terms were significant. All figures were created using GraphPad Prism (United States).

## 3 Results

### 3.1 Effect of quercetin-loaded Fe3O4-NPs on growth performance of *Clostridium perfringens* infected broiler chickens

- The selected growth performance parameters are summarized in Table 3. Prior to *C. perfringens* infection (during starter and grower periods), BWG and FCR were significantly improved in broilers fed quercetin-loaded Fe_3_O_4_-NPs (*p* < 0.05), followed by those supplemented by either with quercetin or Fe_3_O_4_-NPs alone unlike the control unsupplemented birds. During the overall experimental period (38 days), necrotic enteritis infected birds showed a reduction in growth performance associated parameters and increased mortality rates (38%). In contrast, the group fed the combination of quercetin and Fe_3_O_4_-NPs exhibited the greatest improvement in BWG and FCR (*p* < 0.05) and had the lowest mortality rate (4%).

**Table 3:** Efficacy of dietary supplementation of quercetin or Fe_3_O_4_-NPs alone or their nano composite (quercetin-loaded Fe_3_O_4_-NPs) on growth performance parameters of broilers challenged with *C. perfringens* at grower stage.

	Control	Fe_3_O_4_-NPs	Quercetin	Quercetin-loaded Fe_3_O_4_-NPs	*p* value	SEM
Starter (1–10 days)						
BW (g/bird)	344^d^	354^c^	360^b^	375^a^	<0.001	6.51
BWG (g/bird)	297^d^	309^b^	314^b^	328^a^	<0.001	6.56
FCR	382^b^	385^ab^	386^ab^	390^a^	0.04	9.72
FI (g/bird)	1.29^a^	1.25^b^	1.24^b^	1.18^c^	<0.001	2.71
Grower (11–20 days)						
BW (g/bird)	1076^c^	1247^b^	1337^a^	1358^a^	<0.001	11.42
BWG (g/bird)	733^c^	892^b^	977^a^	983^a^	<0.001	12.56
FCR	1.91^a^	1.68^b^	1.58^c^	1.56^c^	<0.001	0.0016
FI (g/bird)	1401^b^	1501^a^	1547^a^	1535^a^	0.01	14.06
Finisher (21–35 days)						
BW (g/bird)	1964^d^	2300^c^	2327^b^	2553^a^	<0.001	5.34
BWG (g/bird)	888^d^	1052^b^	990^c^	1196^a^	<0.001	2.22
FCR	2.61^a^	1.87^b^	1.92^b^	1.79^c^	<0.001	0.001
FI (g/bird)	2316^a^	1967^c^	1897^c^	2140^b^	<0.001	5.53
Allover (1–35 days)						
BWG (g/bird)	1918^d^	2254^b^	2281^c^	2506^a^	<0.001	12.69
FI (g/bird)	4099^a^	3852^b^	3833^b^	4061^a^	<0.001	4.95
FCR	2.14^a^	1.71^b^	1.68^c^	1.62^d^	<0.001	0.001
Mortality (%)	38.00^a^	14.00^b^	10.00^c^	4.00^d^	<0.001	1.22

Control: birds Fe_3_O_4_d basal diet and challenged with *C. perfringens* at the grower period, Fe_3_O_4_-NPs: birds fed basal diet supplemented with 60 mg/kg diet of Fe_3_O_4_-NPs, quercetin: birds fed basal diet supplemented with 300 mg/kg diet of quercetin, quercetin-loaded Fe_3_O_4_-NPs: birds fed basal diet supplemented with a nanocomposite of quercetin and Fe_3_O_4_-NPs (300 mg/kg diet). BW: body weight; BWG: body weight gain; FI: feed intake; FCR: feed conversion ratio; ^a–d^Mean values with different letters in the same row differ significantly at *p* < 0.05.

### 3.2 Quercetin-loaded Fe3O4-NPs not only reduced *Clostridium perfringens* abundance but also diminished its virulence

**Figure 2:**
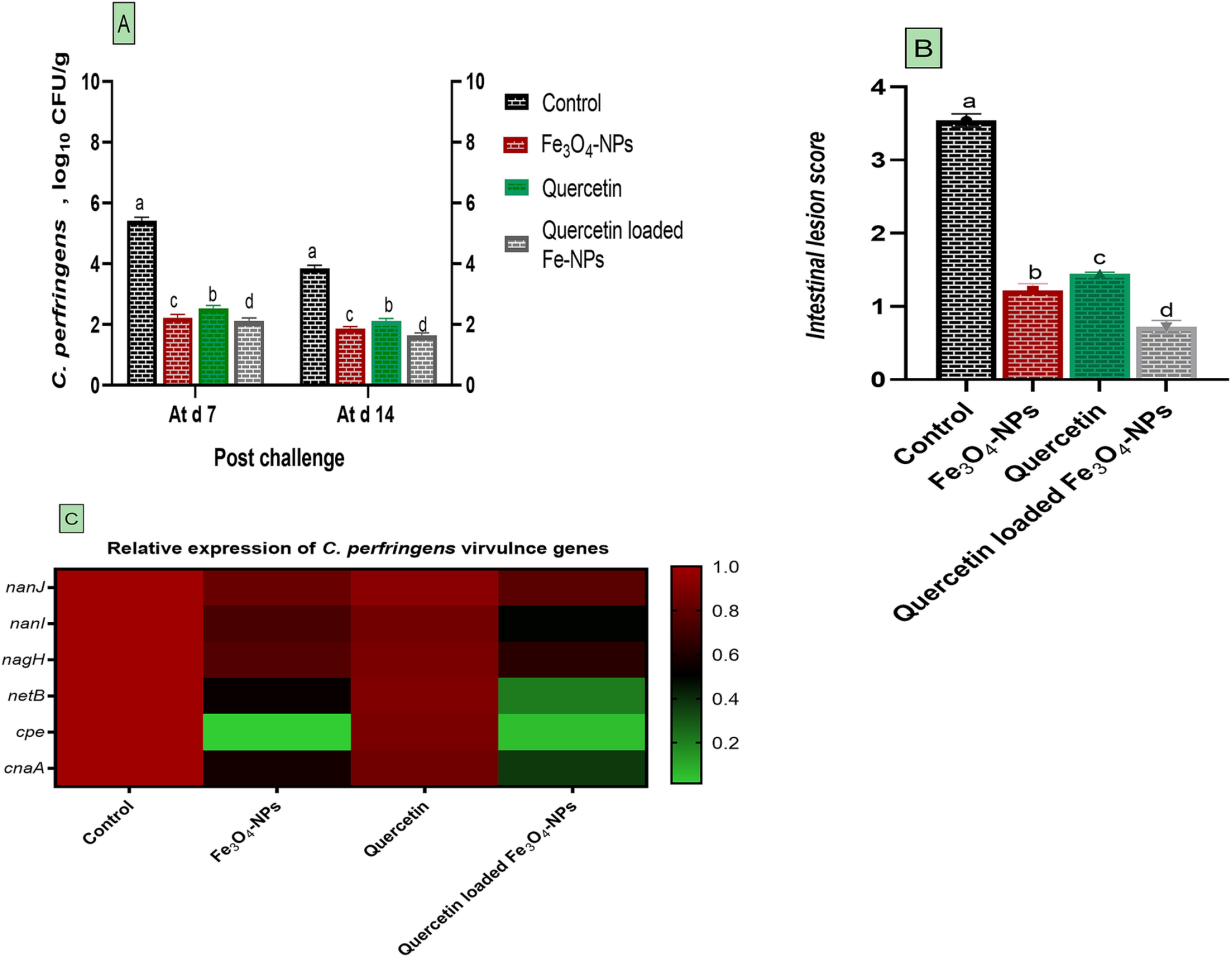
Efficacy of dietary supplementation of quercetin or Fe_3_O_4_-NPs alone or their nano composite (quercetin-loaded Fe_3_O_4_-NPs) on *C. perfringens* population in intestinal contents of broiler chickens at 7- and 14-days post challenge with *C. perfringens* **(A)**, intestinal lesion score **(B)** and the expression of *C. perfringens* virulence genes **(C)** including *nanJ*, *nanI*, *nagH*, *netB*, *cpe* and *cnaA*. Control: birds fed basal diet and challenged with *C. perfringens* at the grower period, Fe_3_O_4_-NPs: birds fed basal diet supplemented with 60 mg/kg diet of Fe_3_O_4_-NPs, quercetin: birds fed basal diet supplemented with 300 mg/kg diet of quercetin, quercetin-loaded Fe_3_O_4_-NPs: birds fed basal diet supplemented with a nanocomposite of quercetin and Fe_3_O_4_-NPs (300 mg/kg diet). ^a–d^ Means within the same column carrying different superscripts are significantly different at *p* < 0.05.

- Results of *C. perfringens* quantification using real-time PCR are presented in Figure 2. The intestinal contents of infected broilers received a basal diet supplemented with Fe_3_O_4_-NPs or quercetin, whether combined or separately, exhibited significant decreases in *C. perfringens* abundance compared to infected birds received control diet at 7 and 14 days post-challenge (*p* < 0.05). Specifically, birds receiving the combination of quercetin and Fe_3_O_4_-NPs, followed by those fed Fe_3_O_4_-NPs alone, displayed lower *C. perfringens* populations comparing with infected birds (1.64, 1.87 and 3.85 log_10_ CFU per gram, respectively) at 14 days post-challenge (*p* < 0.05; Figure 2A). Consistent with the *C. perfringens* count findings, intestinal lesion scores were lower in groups supplemented with Fe_3_O_4_-NPs, quercetin, or the quercetin-loaded Fe_3_O_4_-NPs unlike the *C. perfringens* infected group. The most notable decreases in intestinal scores were detected in the quercetin-loaded Fe_3_O_4_-NPs group, followed by the Fe_3_O_4_-NPs alone group (Figure 2B). Additionally, expression data for *C. perfringens nanJ*, *nanI*, *nagH*, *netB*, *cpe* and *cnaA* virulence genes demonstrated noteworthy downregulatory roles for either Fe_3_O_4_-NPs, quercetin, or their combination (*p* < 0.05; Figure 2C). Interestingly, dietary inclusion of quercetin-loaded Fe_3_O_4_-NPs resulted in the greatest downregulation in *C. perfringens netB*, *cpe* and *cnaA* virulence genes (0.21, 0.05 and 0.37- fold change, respectively).

### 3.3 Quercetin-loaded Fe_3_O_4_-NPs modulated intestinal innate defense and inflammatory response in broilers challenged with *Clostridium perfringens*

**Figure 3:**
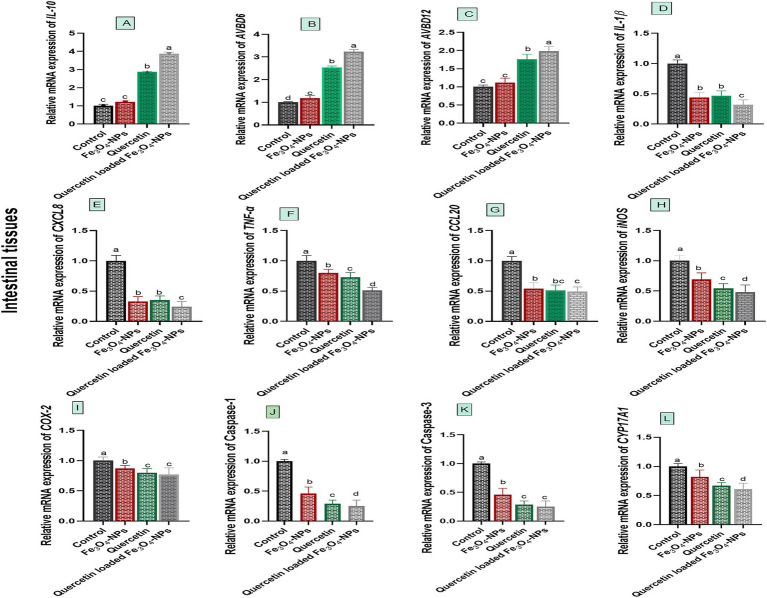
Efficacy of dietary supplementation of quercetin or Fe_3_O_4_-NPs alone or their nano composite (quercetin-loaded Fe_3_O_4_-NPs) on the mRNA expression of host defense antimicrobial peptides (Avian *β*-defensin 6 and 12; *AvBD6*, **B** and *AvBD12*, **C**), inflammatory regulators genes [interleukin, *IL-10*, **(A)**, (*IL*)-*1β*, **(D)**, C-X-C motif chemokine ligand 8; *CXCL8, **E***], tumor necrosis factor-*α* (*TNF*-α, **F**), chemokine C–C motif ligand 20 or also known as macrophage inflammatory proteins −3 α (*CCL20, **G***), inducible nitric oxide synthase (*iNOS*, **H**) cycloox-ygenase-2 (*COX-2, **I***), caspase-1, **(J)**, caspase-3, **K** and cytochrome P450, family 17, subfamily A, polypeptide 1 (*CYP17A1*, **L**) in intestinal tissues of broiler chickens challenged with *C. perfringens.* Control: birds fed basal diet and challenged with *C. perfringens* at the grower period, Fe_3_O_4_-NPs: birds fed basal diet supplemented with 60 mg/kg diet of Fe_3_O_4_-NPs, quercetin: birds fed basal diet supplemented with 300 mg/kg diet of quercetin, quercetin-loaded Fe_3_O_4_-NPs: birds fed basal diet supplemented with a nanocomposite of quercetin and Fe_3_O_4_-NPs (300 mg/kg diet). ^a–d^ Means within the same column carrying different superscripts are significantly different at *p* < 0.05.

Data presented in Figure 3 showed varying expression levels of intestinal innate defense and inflammatory associated markers following *C. perfringens* challenge, in response to supplementation of Fe_3_O_4_-NPs, quercetin, or their combination. Notably, the most marked upregulation levels of intestinal innate antimicrobial defense encoded genes; *AVBD6* and *AVBD12* as well as the anti-inflammatory *IL-10* gene were noted in group receiving quercetin-loaded Fe_3_O_4_-NPs post *C. perfringens* challenge (3.23, 1.98 and 3.78-fold change, respectively).

Relative mRNA expression levels of *IL-1β* and *CXCL8* genes were significantly downregulated (*p* < 0.05) in birds fed quercetin-loaded Fe_3_O_4_-NPs, followed by those fed quercetin or Fe_3_O_4_-NPs alone, unlike the infected control group. Birds supplemented with the combination of quercetin and Fe_3_O_4_-NPs also showed the most pronounced downregulation of *TNF-α* and *iNOS* genes (0.51 and 0.48-fold change, respectively). Additionally, the most notable downregulation of *COX-2* gene expression was observed in the groups fed quercetin and quercetin-loaded Fe_3_O_4_-NPs (0.80 and 0.77-fold change, respectively). For the *CCL20* gene, dietary inclusion of quercetin-loaded Fe_3_O_4_-NPs resulted in detectable downregulation (0.49-fold change) unlike the infected control group (*p* < 0.05).

The relative mRNA expression levels of *CYP17A1*, Caspase-1 and Caspase-3 genes were significantly downregulated (*p* < 0.05) in birds fed quercetin-loaded Fe_3_O_4_-NPs, quercetin, and Fe_3_O_4_-NPs alone, compared to the infected group. Interestingly, *CYP17A1* and Caspase-1 genes achieved their significant lowest expression levels (*p* < 0.05) in birds fed quercetin-loaded Fe_3_O_4_-NPs. Meanwhile, dietary inclusion of quercetin and quercetin-loaded Fe_3_O_4_-NPs resulted in prominent downregulation of the Caspase-3 gene unlike the infected control group (*p* < 0.05), with no significant variations between the quercetin and quercetin-loaded Fe_3_O_4_-NPs groups (*p* > 0.05).

### 3.4 Quercetin-loaded Fe_3_O_4_-NPs promoted antioxidant regulatory intestinal response in broilers

**Figure 4:**
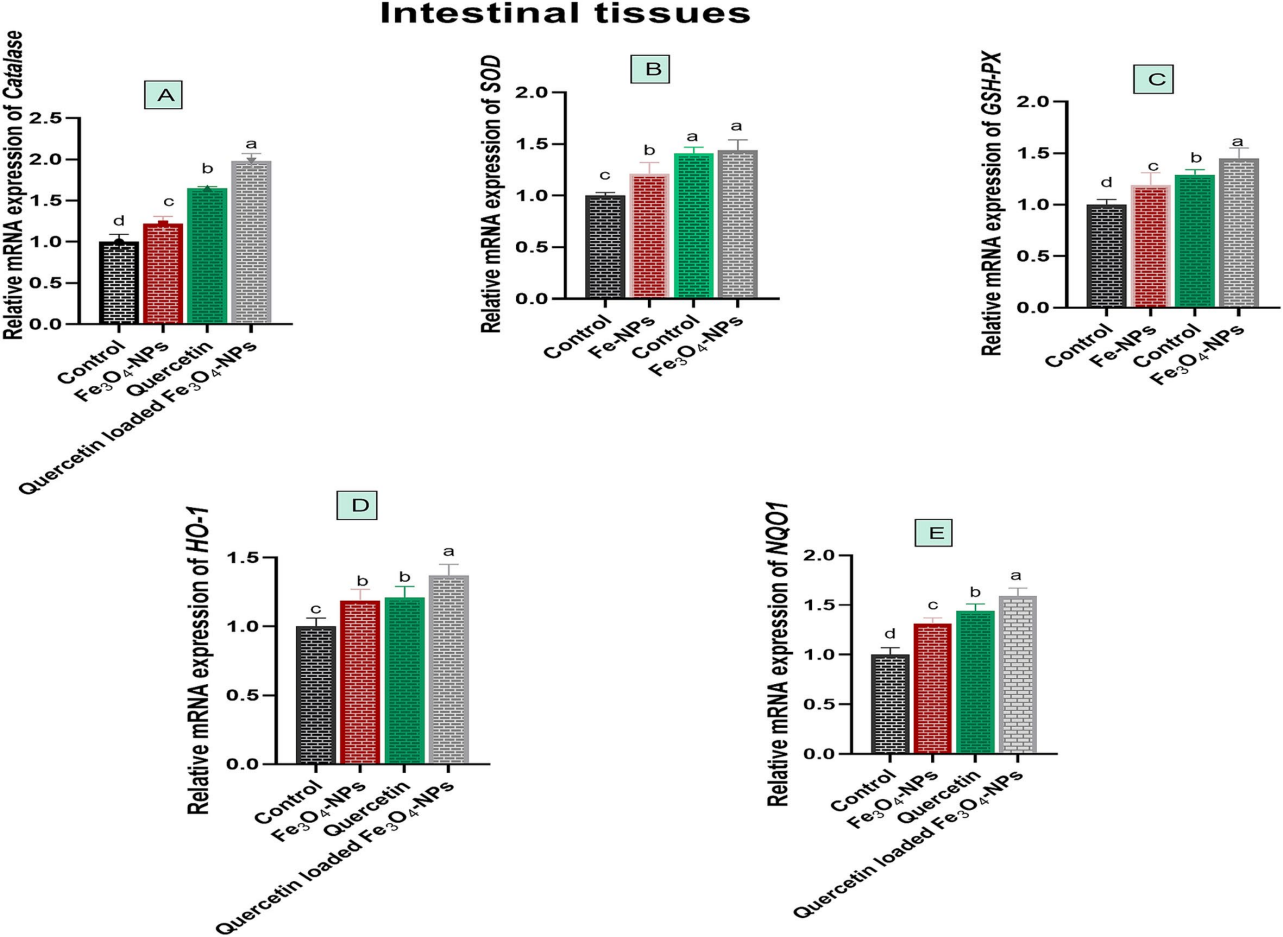
Efficacy of dietary supplementation of quercetin or Fe_3_O_4_-NPs alone or their nano composite (quercetin-loaded Fe_3_O_4_-NPs) antioxidant related genes including catalase **(A)**, superoxide dismutase (*SOD,* **B**), glutathione peroxidase (*GSH*-*Px,* **C**), heme oxygenase-1 (*HO*-*1,* **D**), NAD(P)H dehydrogenase quinone 1 (*NQO1,* **E**) in intestinal tissues of broiler chickens challenged with *C. perfringens.* Control: birds fed basal diet and challenged with *C. perfringens* at the grower period, Fe_3_O_4_-NPs: birds fed basal diet supplemented with 60 mg/kg diet of Fe_3_O_4_-NPs, quercetin: birds fed basal diet supplemented with 300 mg/kg diet of quercetin, quercetin-loaded Fe_3_O_4_-NPs: birds fed basal diet supplemented with a nanocomposite of quercetin and Fe_3_O_4_-NPs (300 mg/kg diet). ^a–d^ Means within the same column carrying different superscripts are significantly different at *p* < 0.05.

Quercetin-loaded Fe_3_O_4_-NPs upregulated antioxidant encoded genes, which consequently mitigated oxidative stress associated with *C. perfringens* infection, as illustrated in Figure 4. The relative mRNA expression levels of antioxidant related genes, including *CAT*, *SOD*, *HO-1*, *GSH-Px* and *NQO1* were significantly increased in birds fed Fe_3_O_4_-NPs, quercetin, or their combination in comparison with the control group. Of note, the most significant increases (*p* < 0.05) in the relative mRNA expression levels of *CAT*, *GSH-Px* and *NQO1* genes were noticed in birds supplemented with quercetin-loaded Fe_3_O_4_-NPs (1.65, 1.45 and 1.59-fold change, respectively). Meanwhile, there was no significant variations (*p* > 0.05) in the transcription levels of the *SOD* gene between the quercetin and quercetin-loaded Fe_3_O_4_-NPs groups (1.41 and 1.44-fold change, respectively). Moreover, the quercetin-loaded Fe_3_O_4_-NPs group showed significant upregulation (*p* < 0.05) of the *HO-1* gene expression level (1.37-fold change), followed by the quercetin (1.21-fold change) and Fe_3_O_4_-NPs (1.19-fold change) groups, compared to the control group. There were no significant differences (*p* > 0.05) between the quercetin and Fe_3_O_4_-NPs groups in terms of *HO-1* expression.

### 3.5 Quercetin-loaded Fe_3_O_4_-NPs promoted antioxidant stability in the breast meat of broilers

**Figure 5:**
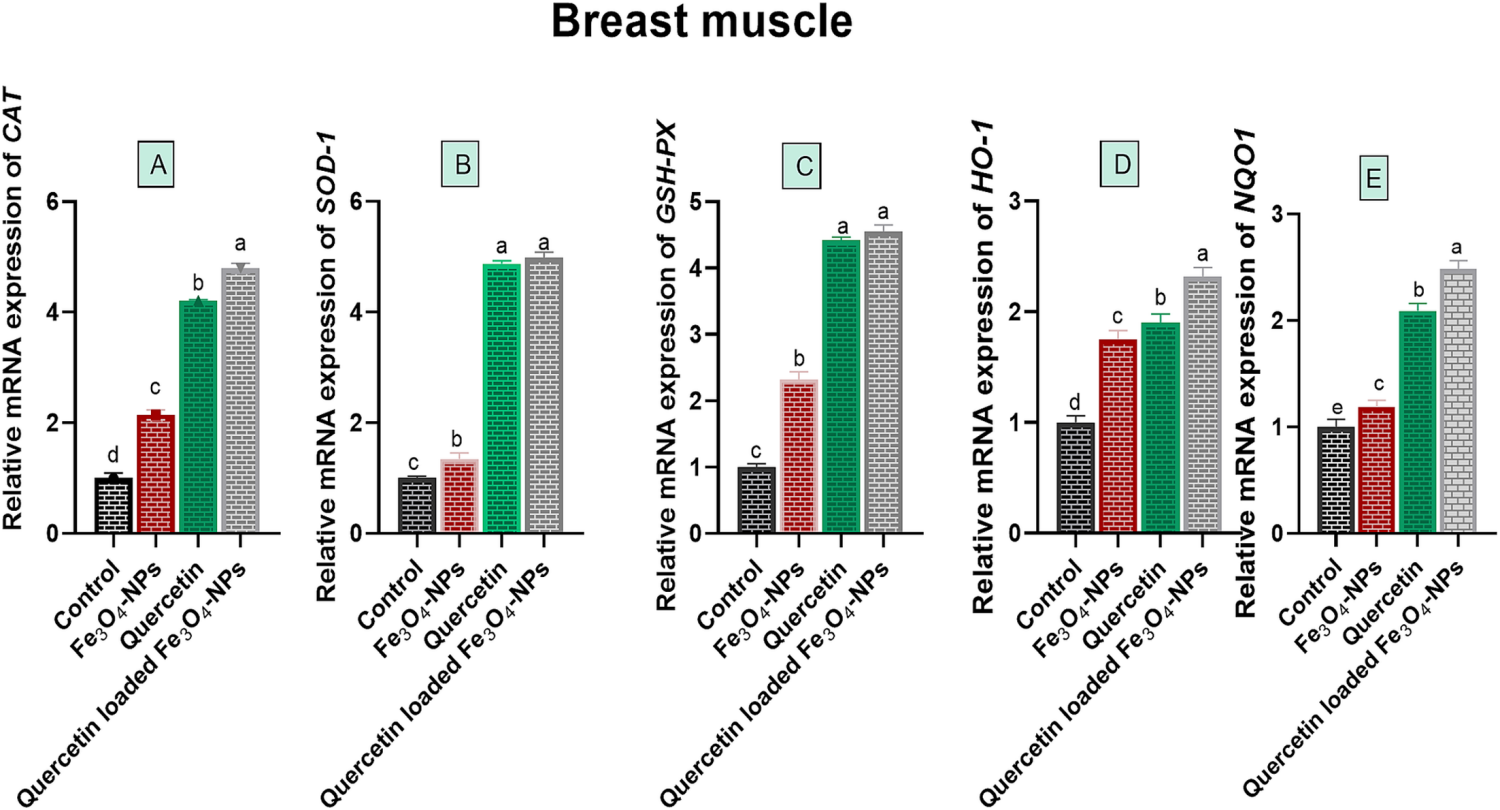
Efficacy of dietary supplementation of quercetin or Fe_3_O_4_-NPs alone or their nano composite (quercetin-loaded Fe_3_O_4_-NPs) antioxidant related genes Catalase **(A)**, superoxide dismutase (*SOD* **B**), glutathione peroxidase (*GSH*-*Px,* **C**), heme oxygenase-1 (*HO*-*1,* **D**), NAD(P)H dehydrogenase quinone 1 (*NQO1,* **E**) in breast muscle tissues of broiler chickens challenged with *C. perfringens*. Control: birds fed basal diet and challenged with *C. perfringens* at the grower period, Fe_3_O_4_-NPs: birds fed basal diet supplemented with 60 mg/kg diet of Fe_3_O_4_-NPs, quercetin: birds fed basal diet supplemented with 300 mg/kg diet of quercetin, quercetin-loaded Fe_3_O_4_-NPs: birds fed basal diet supplemented with a nanocomposite of quercetin and Fe_3_O_4_-NPs (300 mg/kg diet). ^a–d^ Means within the same column carrying different superscripts are significantly different at *p* < 0.05.

Biochemical analysis of broiler breast muscles for oxidative stress-associated enzymes showed a significant reduction in ROS, H_2_O_2_ and MDA levels, with a prominent increase in T-AOC levels in all supplemented broiler groups compared to the control group fed the basal diet (*p* < 0.05; Table 4). Notably, the T-AOC level reached its peak (3.39 U/mg of protein), while ROS, H_2_O_2_, and MDA levels attained their lowest values (32.11 μL/ g tissue, 16.24 μmoL/g tissue and 1.82 nmoL/g tissue, respectively) in the quercetin-loaded Fe_3_O_4_-NPs fed group. Concerning the expression data of antioxidant-related genes in the breast meat of broilers, as shown in Figure 5, there were noticeable increases in the relative mRNA expression levels of antioxidant related genes, including *CAT*, *SOD*, *HO-1*, *GSH-Px* and *NQO1*, in the breast muscles of broilers in response to supplementation of Fe_3_O_4_-NPs, quercetin, or their combination, compared with the control group (*p* < 0.05). Remarkably, the highest transcription levels of *SOD* and *GSH-Px* genes were observed in the quercetin and quercetin-loaded Fe_3_O_4_-NPs fed groups, with no significant differences between them. Moreover, the maximum significant upregulation (*p* < 0.05) of *CAT*, *HO-1* and *NQO1* genes was attained in the quercetin-loaded Fe_3_O_4_-NPs supplemented group with fold changes of 4.79, 2.32 and 2.48-, respectively.

**Table 4:** Efficacy of dietary supplementation of quercetin or Fe_3_O_4_-NPs alone or their nano composite (quercetin-loaded Fe_3_O_4_-NPs) on antioxidant related biomarkers muscle tissues of broilers challenged with *C. perfringens* at grower stage.

	Control	Fe_3_O_4_-NPs	Quercetin	Quercetin-loaded Fe_3_O_4_-NPs	*p* value	SEM
T-AOC (U/mg of protein)	1.67^d^	2.52^c^	2.95^b^	3.39^a^	<0.001	6.51
H_2_O_2_ (μmoL/g tissue)	21.78^a^	19.27^b^	18.25^c^	16.24^d^	<0.001	6.56
ROS (μL/g tissue)	86.10^a^	39.30^b^	32.89^c^	32.11^d^	0.04	9.72
MDA (nmoL/g tissue)	3.95^a^	2.60^b^	2.10^c^	1.82^d^	<0.001	2.71

Control: birds fed basal diet and challenged with *C. perfringens* at the grower period, Fe_3_O_4_-NPs: birds fed basal diet supplemented with 60 mg/kg diet of Fe_3_O_4_-NPs, quercetin: birds fed basal diet supplemented with 300 mg/kg diet of quercetin, quercetin-loaded Fe_3_O_4_-NPs: birds fed basal diet supplemented with a nanocomposite of quercetin and Fe_3_O_4_-NPs (300 mg/kg diet). T-AOC: total antioxidant capacity; H_2_O_2_: hydrogen peroxide; ROS: reactive oxygen species; MDA: malondialdehyde. SEM: standard error of the mean. Means with different superscripts within the same row differ significantly (*p* < 0.05).

### 3.6 Quercetin-loaded Fe_3_O_4_-NPs modulated the hematological picture and immune response

Compared with *C. perfringens challenged group*, significant (*p* < 0.05) increases in RBCs counts and hemoglobin (Hb) concentrations were noted in groups supplemented with quercetin-loaded Fe_3_O_4_-NPs followed by Fe_3_O_4_-NPs (Table 5). Our data indicated that feeding broilers Fe_3_O_4_-NPs, quercetin, or quercetin-loaded Fe_3_O_4_-NPs had an immunomodulatory effect on phagocytes derived from peripheral blood mononuclear cells (Table 5). The most significant enhancements in the phagocytic and killing activities of phagocytes, along with highest lysozyme activity and lowest production of inflammatory serum NO, were noticed in the group of chickens fed quercetin-loaded Fe_3_O_4_-NPs (*p* < 0.05).

**Table 5:** Efficacy of dietary supplementation of quercetin or Fe_3_O_4_-NPs alone or their nano composite (quercetin-loaded Fe_3_O_4_-NPs) on hematological picture and innate immune defense of broilers challenged with *C. perfringens* at grower stage.

	Control	Fe_3_O_4_-NPs	Quercetin	Quercetin-loaded Fe_3_O_4_-NPs	*p* value	SEM
RBCs (×10^6^/μL)	1.89^c^	2.40^a^	2.35^b^	2.43^a^	<0.001	0.94
Hemoglobin (g/dl)	10.54^c^	11.98^a^	11.36^b^	12.10^a^	<0.001	2.11
Phagocytic activity	16.04^d^	18.06^c^	28.03^b^	31.09^a^	<0.001	6.51
Killing efficiency	36.33^d^	38.33^c^	44.67^b^	50.09^a^	<0.001	6.56
Lysozymes (U/ml)	107.33^c^	122.33^b^	119.33^b^	176.33^a^	0.04	9.72
Nitric oxide (μmol/L)	10.76^a^	7.82^b^	5.18^c^	3.61^d^	<0.001	2.71

Control: birds fed basal diet and challenged with *C. perfringens* at the grower period, Fe_3_O_4_-NPs: birds fed basal diet supplemented with 60 mg/kg diet of Fe_3_O_4_-NPs, quercetin: birds fed basal diet supplemented with 300 mg/kg diet of quercetin, quercetin-loaded Fe_3_O_4_-NPs: birds fed basal diet supplemented with a nanocomposite of quercetin and Fe_3_O_4_-NPs (300 mg/kg diet). RBCs, red blood cells. SEM: standard error of the mean. Means with different superscripts within the same row differ significantly (*p* < 0.05).

### 3.7 Quercetin-loaded Fe_3_O_4_-NPs restored broilers’ microbial populations

**Figure 6:**
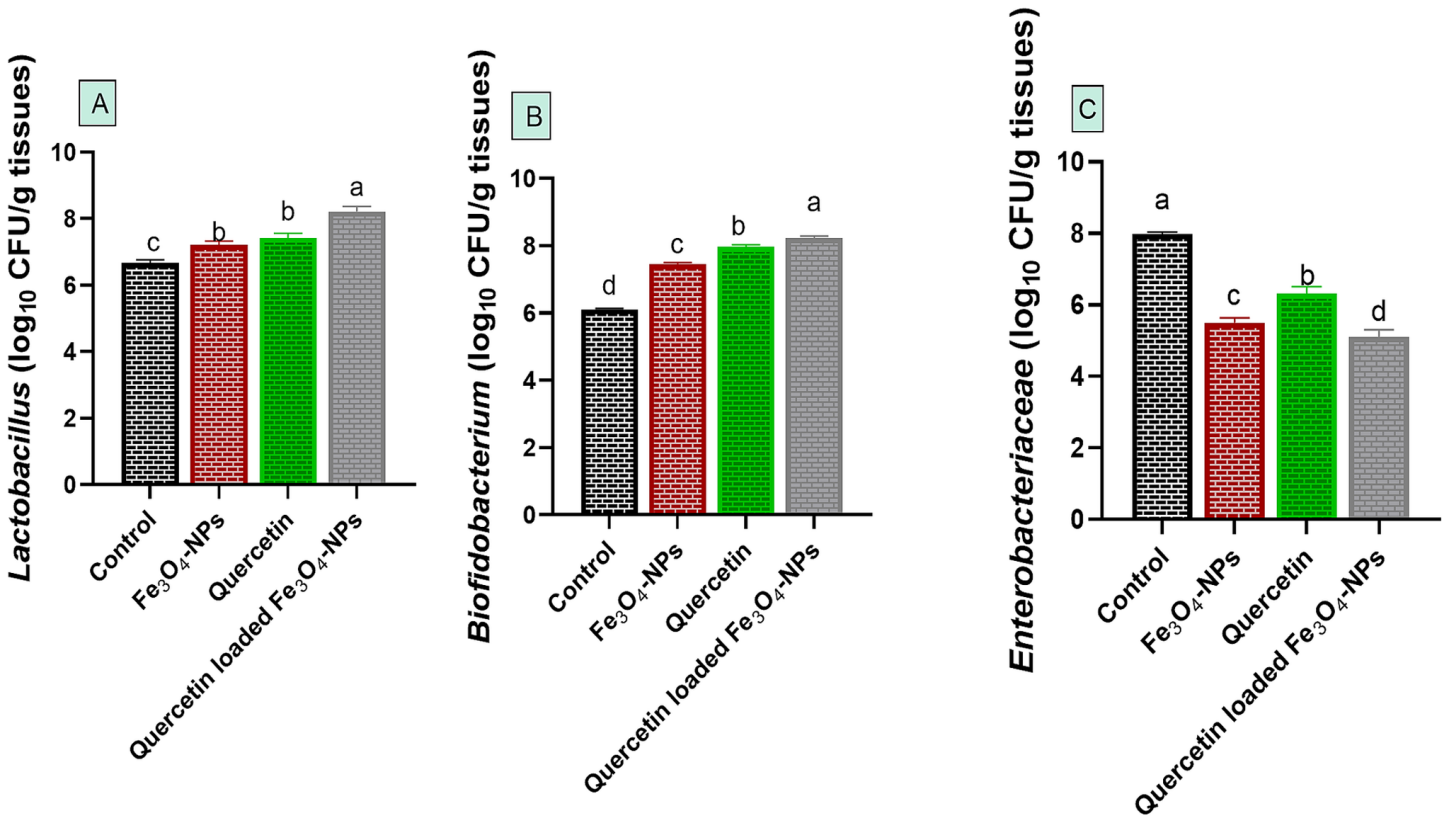
Efficacy of dietary supplementation of quercetin or Fe_3_O_4_-NPs alone or their nano composite (quercetin-loaded Fe_3_O_4_-NPs) on intestinal *Lactobacillus* spp. **(A)**, *Bifidobacterium* spp. **(B)** and *Enterobacteriaceae* **(C)** populations of broiler chickens challenged with *C. perfringens*. Control: birds fed basal diet and challenged with *C. perfringens* at the grower period, Fe_3_O_4_-NPs: birds fed basal diet supplemented with 60 mg/kg diet of Fe_3_O_4_-NPs, quercetin: birds fed basal diet supplemented with 300 mg/kg diet of quercetin, quercetin-loaded Fe_3_O_4_-NPs: birds fed basal diet supplemented with a nanocomposite of quercetin and Fe_3_O_4_-NPs (300 mg/kg diet). ^a–d^ Means within the same column carrying different superscripts are significantly different at *p* < 0.05.

Notably, *C. perfringens* intestinal colonization during infection in control infected group was associated with microbial disruption, as reflected by a notable decrease in the populations of *Lactobacillus* and *Bifidobacterium* species, alongside an increase in the *Enterobacteriaceae* community. Meanwhile, supplementation with quercetin-loaded Fe_3_O_4_-NPs restored this microbial balance by favoring the growth of beneficial *Lactobacillus* and *Bifidobacterium* species while reducing the abundance of harmful *Enterobacteriaceae* (Figure 6).

### 3.8 Histopathological examination

**Figure 7:**
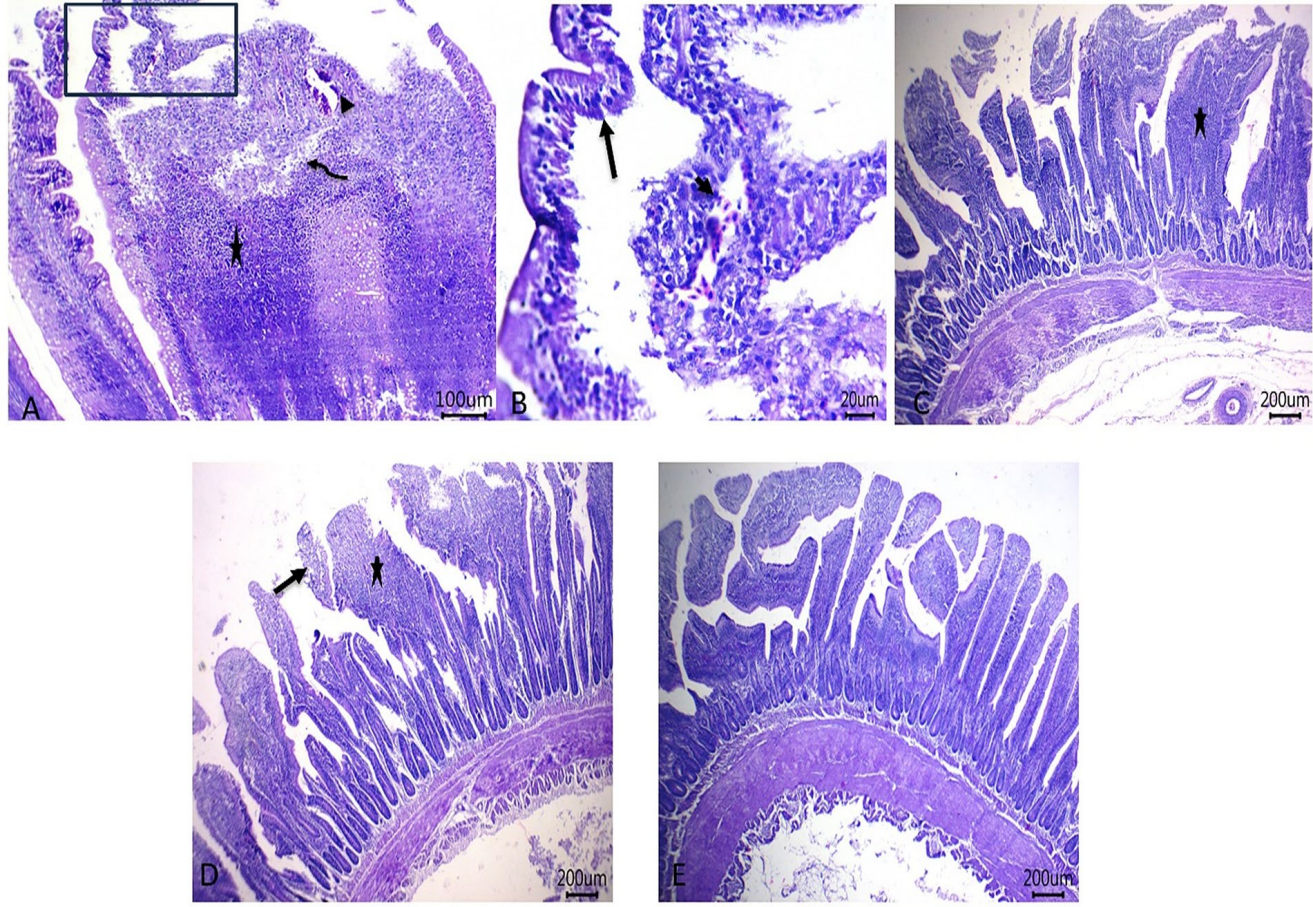
Photomicrograph of H&E stained intestinal sections showing sloughing of some mucosal epithelium and necrotic sheets of lining epithelium with pyknotic nuclei (arrow), marked inflammatory cells’ infiltrations within lamina propria and submucosa (star) adjacent to the area of lytic necrosis (curved arrow) besides the presence of dilated blood vessels (arrowheads) in control infected birds **(A,B)**, mild aggregations of inflammatory cells within mucosal and submucosal layers (star) in Fe_3_O_4_-NPs supplemented birds **(C)**, necrotic and sloughed area at some mucosal villi (arrow) with moderate numbers of inflammatory cells’ infiltration within lamina propria and submucosa (star) **(D)** and maintenance of mucosal villi, lamina propria, mucosal crypts and musculosa in quercetin-loaded Fe_3_O_4_-NPs fed birds **(E)**, scale bar 200,100 and 20 *μ*m. Control: birds fed basal diet and challenged with *C. perfringens* at the grower period, Fe_3_O_4_-NPs: birds fed basal diet supplemented with 60 mg/kg diet of Fe_3_O_4_-NPs, quercetin: birds fed basal diet supplemented with 300 mg/kg diet of quercetin, quercetin-loaded Fe_3_O_4_-NPs: birds fed basal diet supplemented with a nanocomposite of quercetin and Fe_3_O_4_-NPs (300 mg/kg diet).

**Figure 8:**
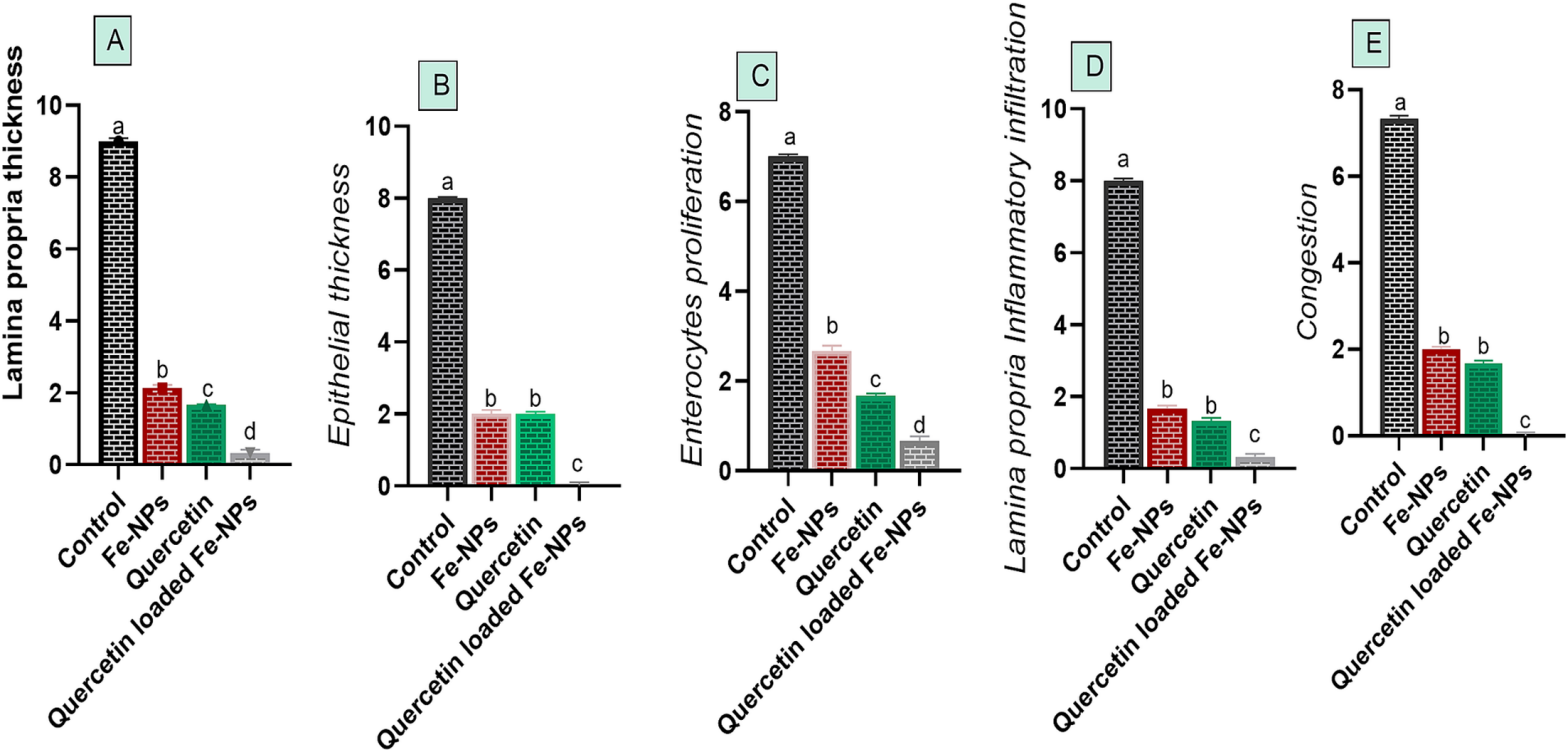
Efficacy of dietary supplementation of quercetin or Fe_3_O_4_-NPs alone or their nano composite (quercetin-loaded Fe_3_O_4_-NPs) on metric evaluation of the intestinal tissues alterations **(A–E)** of broilers challenged with challenged with *C. perfringens* at grower stage. Control: birds fed basal diet and challenged with *C. perfringens* at the grower period, quercetin: birds fed basal diet supplemented with (300 mg/kg diet of quercetin, Fe_3_O_4_-NPs: birds fed basal diet supplemented with 60 mg/kg diet of Fe_3_O_4_-NPs, quercetin-loaded Fe_3_O_4_-NPs: birds fed basal diet supplemented with a nanocomposite of quercetin and Fe_3_O_4_-NPs; 300 mg/kg diet). ^a–d^Means within the same column carrying different superscripts are significantly different at *p* < 0.05.

Histopathological pictures of intestinal tissues from broilers experimentally infected with *C. perfringens*, in response to supplementation of Fe_3_O_4_-NPs, or quercetin, or formulated quercetin-loaded Fe_3_O_4_-NPs, are illustrated in Figure 7. Infected control birds revealed sloughing of some mucosal epithelium and necrotic sheets of lining epithelium with pyknotic nuclei. Furthermore, there were marked infiltrations of inflammatory cells within the lamina propria and submucosa adjacent to areas of lytic necrosis, as well as the presence of dilated blood vessels. Moreover, birds supplemented with Fe_3_O_4_-NPs displayed mild aggregations of inflammatory cells within the mucosal and submucosal layers. Quercetin-fed birds exhibited necrotic and sloughed areas on some mucosal villi, with moderate infiltration of inflammatory cells in the lamina propria and submucosa. Notably, the inclusion of quercetin-loaded Fe_3_O_4_-NPs restored the intestinal histopathological architecture, preserving mucosal villi, lamina propria, mucosal crypts, and musculosa. *C. perfringens* infected birds had the highest ileal total scores due to increased epithelial and lamina propria thickness from inflammatory cell infiltration, congestion, and excessive enterocyte proliferation. Conversely, supplementation with quercetin-loaded Fe_3_O_4_-NPs subsided these exaggerated ileal scores, as reflected by improvements in the measured metrics (Figure 8).

## 4 Discussion

Necrotic enteritis caused by *C. perfringens* infection in broilers is a multifactorial disease influenced by various predisposing factors (29). This disease poses a significant threat not only to the health of broilers but also to the profitability of the poultry industry, particularly due to the short rearing period of broilers (6). The disease is also a public health concern due to its potential as a foodborne infection (1). The ban forbidding antibiotic use in poultry to combat bacterial resistance caused a spike in the occurrence of NE in broiler populations. The use of novel generations of nanocomposite based on natural products in poultry diet seems to be promising (30, 31). Quercetin is a natural flavonoid with pleotropic biological effects. It has been demonstrated that quercetin supplementation mitigates intestinal inflammation and maintains intestinal immune barrier integrity in aged laying hens (15). Iron oxide nanoparticles, particularly magnetite NPs, have revolutionized various fields due to their high surface area, availability, cost-effectiveness, rapid adsorption kinetics, and ease of dispersibility in solutions (32). Additionally, their superparamagnetic propderties allow for easy separation from reaction mixtures using a simple magnet. In this context, incorporation of quercetin into the magnetite NPs in a suitable pharmaceutical formula, that overcomes their low bioavailability and maintains their action, would be required. Moreover, the exact mechanism of their targeted action and their effectiveness in enhancing broiler resistance to *C. perfringens* infection needs to be further explored. Therefore, our study explored the immunomodulatory and protective effect of magnetite NPs enclosed quercetin on broiler chickens experimentally infected with *C. perfringens*.

The data in this study indicated that broilers challenged with *C. perfringens* and received quercetin-loaded Fe_3_O_4_-NPs showed a restored BWG and FCR over the whole experimental period. In consistence with these results, Goliomytis, Tsoureki, Simitzis, Charismiadou, Hager-Theodorides and Deligeorgis (17) demonstrated that dietary levels of free dietary quercetin (0.5 g/kg of feed) enhanced FCR of broiler chickens. Additionally, the production efficiency of broiler chickens was enhanced by supplemental quercetin at a concentration of 200 ppm (33). The augmented growth performance of broilers post dietary supplementation of nanosized particles of quercetin at lower levels (100 ppm/kg) is attributed to their enhanced bioavailability and solubility (34). Additionally, quercetin supplementation positively effect on growth performance and nutrient digestibility of broilers (16). Moreover, supplementing nano sized magnetite iron exerted a notable growth promoting role during the entire production cycle, as evidenced by increased body weight gain and improved FCR in broilers. These favorable findings can be attributed to the critical role of iron in oxygen transport, the regulation of various proteins and enzymes involved in cell development and differentiation, and overall health maintenance (32). Besides, it participate pointedly to the tricarboxylic acid cycle via supporting many enzymes, which assist in the elimination of harmful metabolites by peroxidases and catalases within iron (35). Furthermore, iron nanoparticles at the level of 40 mg/kg increased broiler performance (36). This may be due to the ability of nanosized particles to cross biological barriers more effectively, be rapidly absorbed by cells, and thus demonstrate better bioavailability compared to other mineral forms (37). Specifically, encapsulating potent antioxidant bioactive components such as quercetin within Fe₃O₄-NPs in broiler feed provides additional protection and allows for synergistic effects that enhance bird performance. Furthermore, conjugating quercetin with superparamagnetic iron oxide nanoparticles improves its bioavailability compared to free quercetin (38).

In addition, our pathological images showed that *C. perfringens* infection in broilers caused multifocal areas of necrosis, marked inflammatory cells’ infiltrations within lamina propria and submucosa adjacent to the area of lytic necrosis besides the presence of dilated blood vessels in intestinal tissues with high scored histological alterations. However, the severity of intestinal lesions and histological scores improved following supplementation with quercetin-loaded iron nanocomposites, as indicated by a significant reduction in the mRNA expression of netB and cpe toxin-related gene. In this regard, NetB toxin is considered a pore forming toxin regulated by *netB* gene and played a role in progression of the histopathological lesions of NE infected chickens (39). This inflammatory necrosis induced by *C. perfringens* toxin occurs through the formation of mushroom-like transmembrane pores in enterocytes. This process is mediated by bacterial adhesion to the intestinal mucosa via fimbrial adhesins (40). Consequently, these transmembrane pores disrupt the cell membrane’s osmotic balance, leading to osmotic cell lysis and necrosis (41). Similar to NetB toxin, Cpe enterotoxin also induces multiple pore formation in the plasma membrane of epithelial cells by binding to the claudin receptor. This leads to calcium influx, activation of calpain and caspase-3, and ultimately, cell death (41). Accordingly, Kumar and his colleagues studied the antimicrobial effect of microencapsulated formula containing bioactive natural molecules on Cobb birds infected with both Eimeria and *C. perfringens*. They demonstrated restoration in duodenal histopathological architecture in birds that were fed this microencapsulated formula (6).

Enterocytes are known to play a key role in regulating iron absorption and its transport into circulation (41, 42). The current study suggested that necrotic damage to enterocytes impairs absorption of iron. This, in turn, induced an oxidative stress response due to the accumulation of free radicals. Therefore, supplementing poultry diets with nano-iron combined with natural flavonoids such as quercetin enhances iron absorption and bioavailability, helping enterocytes better tolerate oxidative damage induced by *C. perfringens* infection. Our data indicated that the improvement in growth performance was associated with a reduction in the severity of intestinal inflammation and necrosis caused by *C. perfringens* infection in broilers.

Although it has been reported that the CFU of *C. perfringens* can reach 10^5^ in healthy chickens, predisposing factors affecting the gut microenvironment and host immunity can lead to higher colonization and increased toxin production, contributing to necrotic enteritis (NE) in birds (3). A comparative study of biologically and chemically synthesized iron oxide nanoparticles showed that biologically synthesized nanoparticles, which contained natural plant extracts, enhanced the microbicidal activity of the nanoparticles against *S. aureus* and *P. aeruginosa* strains isolated from poultry feed (21). However, the direct antimicrobial activity of quercetin-loaded iron nanocomposite against *C. perfringens* isolated from broilers, which consequently hinders its colonization within chicken’s intestine is not deeply investigated until yet. Our data reported that infected control birds expressed higher intestinal *C. perfringens* populations with exalted *C. perfringens* virulence. Conversely, broilers fed quercetin-loaded Fe_3_O_4_-NPs exhibited a reduction in intestinal *C. perfringens* abundance along with diminishing in the mRNA expression of *nanJ*, *nanI*, *nagH* and *cnaA* virulence genes. Previous studies have proven the antimicrobial activities of magnetic iron against many bacterial and parasitic strains (43, 44). Additionally, incorporating an optimal amount of the powerful antioxidant quercetin into Fe₃O₄-NPs enhanced the host’s endogenous defense mechanisms against microbial infections and external stressors (45).

Likewise, the majority of magnetic NPs exerts a bactericidal character as applied against both Gram positive and Gram negative bacteria (46). Moreover, iron oxide magnetic nanoparticles can be used as antimicrobial delivery systems due to their enhanced ability to deliver antimicrobials specifically to targeted site (47) without using of antibiotics (48). Furthermore, magnetite iron oxide NPs were proven to have a strong antibacterial activity through numerous theories as following: iron NPs small size enables them to penetrate the bacterial cell wall triggering cell membrane disruption (49) and oxidative cytotoxic damage to bacterial cell via elevating the bacterial cells reactive hydroxyl radicals (50) g (70). Additionally, they can bind to bacterial DNA and proteins, thereby initiating a bactericidal effect (51) besides the high magnetite role of small sized nanoparticles on bacterial cells causing clogging between bacterial cells instigating their death (52). Altogether, proteolytic, hydrolytic and collagenolytic enzymes like NagH, NanI and NanJ secreted from *C. perfringens* promote extracellular matrix and intercellular damage, mucosal permeability and cell death (1). Moreover, upregulation of *cnaA* gene associated with collagen adhesion protein was reported to facilitate *C. perfringens* adhesion to extracellular matrix and enhance their colonization within ileal mucosa (71). In this context, iron oxide nanoparticles exhibit exceptional paramagnetic activity, generating magnetic fields with strong adhesion properties and further cause biofilm destruction and cell death through mechanisms such as vibration damage, local hyperthermia, cell fusion, membrane rupture, and ultimately, cell death (53). Furthermore, iron oxide nanocrystals can prohibit clostridial spore germination and diminish associated inflammation (54).

In addition, quercetin can inhibit the exaggerated inflammatory response following infection via subsiding the expression of *TNF-α*, *IL-1β*, and *IL-6* in macrophages (55). Moreover, dietary supplementation of quercetin improved immune function by increasing the secretion of immune molecules to protect against infection (56). Also, quercetin exerts broad-spectrum antibacterial properties via disrupting cell walls of bacteria, changing the bacterial cells permeability; impeding the synthesis of nucleic acids, and in that way impacting the synthesis and expression of proproteins; and decreasing enzyme and toxin activities (57). Collectively, the prominent antibacterial effect proved for iron oxide nanoparticles combined with quercetin was attributed to their destructive effect on bacterial cell wall and subsequently losing their different virulence factors enabling them to establish an infection and damage the host and interfering with host immunity (58). These previous findings further supported the superior role of combining both quercetin and iron nanoparticles on broilers performance and fight against *C. perfringens infection.*

Besides the above-mentioned issues, quercetin-loaded Fe_3_O_4_-NPs improved antioxidant stability in broilers breast muscles through regulation of antioxidant enzymes encoded genes. Similar findings reported by Goliomytis and his co-workers, where inclusion of quercetin (0.5 and 1 g/kg of feed) to broiler diets for 42 days indicated improvement in growth performance and oxidative stability of broilers breast muscles (17). Coordinated with previously published data, a study reported that using nano-Fe (40 mg/kg) either alone or combined with a natural extract in broilers’ diets improved their resistance to heat stress. The study proposed that nano-Fe enhanced broiler growth performance and meat quality associated with low mortality under hot conditions (36). Similarly, quercetin-loaded Fe_3_O_4_-NPs reduced oxidative stress associated with the effect of *C. perfringens* secreted toxins by increasing the mRNA expression of antioxidant-related genes (*CAT, SOD1, GSH-Px, HO-1*, and *NQO1*), compared to the control non-supplemented group. In the same line, upregulation of *HO-1* is a protective feedback mechanism of cells against oxidative stress associated damage (31). On the other hand, the increased production of intestinal avian defensins, due to the upregulation of their related genes (*Avβd6* and *Avβd12*), in *C. perfringens*-infected broilers fed a diet supplemented with quercetin-loaded Fe_3_O_4_-NPs, suggests an indirect bactericidal effect on *C. perfringens*. The increased production of defensins also indicates that quercetin-loaded Fe_3_O_4_-NPs improve epithelial cell function and mucosal integrity (72) reported the ability of quercetin supplementation in boosting gut integrity as evidenced by increases in mRNA expression of genes encoding intestinal tight junction protein; zona occludens-1 (ZO-1) and occludin in broilers challenged model compared to control group fed basal diet only Additionally, quercetin represents one of the natural flavonoids, which could protect intestinal tight junction integrity from oxidative stress through its antioxidant effect. Moreover, quercetin ameliorated intestinal inflammation in broiler chickens through restoring intestinal barrier functions and microbiota community, which consequently reduced inflammatory mediators and mucosal damage (72). Given that *CXCL8* is an inflammatory mediator that has a role in activation and recruitment of neutrophils (73), pro-inflammatory cytokines stimulate *CCL20* production, which has a role in T-cell infiltration (74). These inflammatory mediators exaggerate mucosal damage, vascular permeability, oxygen species production and recruitment of heterophils, monocytes and lymphocytes to inflamed bird’s gut (1). Indeed, the increase in the production of antioxidant enzymes post supplementation of quercetin-loaded Fe_3_O_4_-NPs could reduce COX-2 and iNos production, which consequently reduce inflammation. These findings were supported by downregulation of *TiNF-α*, *IL-1β*, *Caspase-1* and *CXCL8* genes in groups of bird fed quercetin-loaded Fe_3_O_4_-NPs suggesting the neglected effect of NE derived *C. perfringens* virulence on epithelial cells. Furthermore, upregulation of anti-inflammatory mediators; *IL-10* and *HO-1* suggested the ability of quercetin-loaded Fe_3_O_4_-NPs to induce polarization of anti-inflammatory macrophages phenotypes promoting the homeostatic response within intestinal barriers, which come concurrently with (75).

*Clostridium perfringens* α-toxin induces hemolysis of erythrocytes in many species involving poultry (59). In the current study, the RBCs counts and hemoglobin concentration in groups supplemented with Fe_3_O_4_-NPs post *C. perfringens* challenge exhibited higher values unlike un supplemented control challenged group. These outcomes could be attributed to their bactericidal role against experimental challenge beside their impact on augmenting RBCs synthesis (60) which was further boosted following formulation of iron into nano sized molecule (32). We further investigated whether quercetin-loaded Fe₃O₄-NPs, as feed additives in broiler diets, have a systemic effect in preventing microbial spreading or its products, thereby reducing endotoxemia and systemic inflammation. This study demonstrated that broilers fed quercetin-loaded Fe₃O₄-NPs showed enhanced phagocytic capacity and killing ability of phagocytes derived from peripheral blood mononuclear cells, compared to the infected, non-supplemented control group. This activation was associated with increased lysozyme activity and reduced nitric oxide production, suggesting that iron-quercetin may mitigate systemic inflammation caused by *C. perfringens* in peripheral blood. Indeed, Zanella and colleagues proposed that iron oxide nanoparticles can cross lipid bilayers directly, creating transient conductance without adversely affecting membrane integrity (76). This effect may explain the enhanced immunity and antioxidant activity observed in the current study from combining iron nanoparticles with quercetin, particularly in combating inflammatory necrotic enteritis caused by *C. perfringens* infection in broilers.

Mounting evidence indicated the relation between microbiota abundance and NE in chickens (77). For instance, the relation between low abundance of *Ruminococcus* species and NE derived *C. perfringens* was reported (3). Herein, there was an increase in the abundance of *Lactobacillus* and *bifidobacterium* species in quercetin-loaded Fe_3_O_4_-NPs supplemented groups compared to the control one. Accordingly, lactobacillus counts were boosted (*p* < 0.05), due to enhancement of the gut microbiota environment in quercetin-supplemented groups (33).

Lactobacillus and bifidobacteria produce short-chain fatty acids that serve as energy sources for intestinal bacteria, enhance nutrient utilization, improve immune defense, and aid in pathogen clearance from the gut (77, 78). In this context, quercetin alleviated microbiota dysbiosis caused by antibiotics in an animal model of liver damage by promoting the growth of *Lactobacillus*, *Bifidobacterium*, and *Bacteroides*, while reducing the abundance of *Enterobacteriaceae* and *Clostridium* species (79). Moreover, dietary inclusion of optimum level of iron plays an important role in increasing the abundance of beneficial microbiota; however iron deficiency can reduce its load and its overload can augment the growth of harmful ones like *E. coli* and *Bacteroides* (80). Another study reported that feeding broilers on *Lactobacillus* probiotics enhanced broiler resistance against *C. perfringens* through the competitive exclusion mechanisms experimental infection and mitigated inflammatory NE associated with its infection (73).

## 5 Conclusion

The use of quercetin-loaded Fe_3_O_4_-NPs represents a promising new feed additive for broilers’ diets, aligning with the global goal of forbidding the uncontrolled antibiotics use while ensuring efficient and sustainable poultry production. Herein, the newly formulated nanocomposite strengthens intestinal and antioxidant barriers supporting maximized performance during NE experimental model. The association between reduction in *C. perfringens* abundance and its virulence suggests the direct antimicrobial effect of quercetin-loaded iron nanocomposite in potentiating chicken intestinal microenvironment, barrier functions and immunity, thereby hindering *C. perfringens* colonization and its effect on intestinal mucosal damage. Finally, this study strongly directs future researches for expanding the application of other nanomaterials to trigger advances in nanotechnology development particularly, in poultry feed industry.

## Data Availability

The original contributions presented in the study are included in the article/supplementary material. Further inquiries can be directed to the corresponding author.
